# Effect of β-Cyclodextrin on Physicochemical Properties of an Ionic Liquid Electrolyte Composed of *N*-Methyl-*N*-Propylpyrrolidinium bis(trifluoromethylsulfonyl)amide

**DOI:** 10.3389/fchem.2019.00090

**Published:** 2019-02-20

**Authors:** Mio Suzuki, Naoya Kurahashi, Yuko Takeoka, Masahiro Rikukawa, Masahiro Yoshizawa-Fujita

**Affiliations:** Department of Materials and Life Sciences, Sophia University, Tokyo, Japan

**Keywords:** ionic liquids, pyrrolidnium, TFSA, β-cyclodextrin, inclusion complex

## Abstract

Ionic liquids (ILs) are promising electrolyte materials for developing next-generation rechargeable batteries. In order to improve their properties, several kinds of additives have been investigated. In this study, β-cyclodextrin (β-CD) was chosen as a new additive in IL electrolytes because it can form an inclusion complex with bis(trifluoromethylsulfonyl)amide (TFSA) anions. We prepared the composites by mixing *N*-methyl-*N*-propylpyrrolidinium bis(trifluoromethylsulfonyl)amide/LiTFSA and a given amount of triacetyl-β-cyclodextrin (Acβ-CD). The thermal behaviors and electrochemical properties of the composites were analyzed by several techniques. In addition, pulse field gradient NMR measurements were conducted to determine the self-diffusion coefficients of the component ions. The addition of Acβ-CD to the IL electrolytes results in the decrease in the conductivity value and the increase in the viscosity value. In contrast, the addition of Acβ-CD to the IL electrolytes induced an improvement in the anodic stability because of the formation of an inclusion complex between the Acβ-CD and TFSA anions. CDs are potential candidates as additives in IL electrolytes for electrochemical applications.

## Introduction

Ionic liquids (ILs) have been attractive as electrolyte materials because of their unique properties such as high ionic conductivity at room temperature and wide potential window (Armand et al., 2009). In addition, as ILs have a low vapor pressure and low flammability, they will be suitable for developing safer electrolytes instead of organic solvents (Ohno, 2011). Among onium cations, pyrrolidinium-based ILs are primarily being used as electrolytes in rechargeable batteries (Ishikawa et al., 2006; Matsumoto et al., 2006; Seki et al., 2008; Yoon et al., 2015). Pyrrolidinium-based ILs are superior in thermal and electrochemical stability as compared to those of other onium-based ILs. However, it is difficult to realize target ion transport with ions such as lithium ions or sodium ions in ILs, because the component ions of ILs as solvents also migrate along the potential gradient. New designs of ILs that address such drawback has been proposed by many researchers. For example, one candidate is poly(IL)s, which fix cation or anion species on the polymer chain (Yuan et al., 2013; Nishimura and Ohno, 2014; Qian et al., 2017). Another candidate is zwitterions, which have the cation and anion in the same molecule (Yoshizawa et al., 2004; Narita et al., 2006; Yoshizawa-Fujita et al., 2011). Nevertheless, it is still difficult to achieve a high ionic conductivity over 10^−2^ S cm^−1^ at room temperature and a high lithium transference number (*t*_Li+_) over 0.5.

Rechargeable batteries, especially lithium-ion batteries (LIBs), employing ILs as the electrolyte materials have been developed (Ishikawa et al., 2006; Matsumoto et al., 2006; Seki et al., 2008; Ohno, 2011). For practical applications, a high energy density of LIBs is required. In order to improve the energy density of LIBs, the cells are needed to be operated at higher cut-off voltages. However, high cut-off voltages induce a significant decrease in the charge/discharge cycle stability of LIBs due to the decomposition of electrolytes. As a result, a passivation layer on the electrode is formed, even when ILs are used as electrolytes (Seki et al., 2008). The decomposition reaction of electrolytes should be suppressed at high cut-off voltages to allow the use of high-voltage cathode materials [e.g., LiCo_1/3_Ni_1/3_Mn_1/3_O_2_ (Yabuuchi and Ohzuku, 2003), LiNi_0.5_Mn_1.5_O_4_ (Zhu et al., 2014)]. Various additives have been used to improve the anodic stability of electrolyte materials (Franco, 2015).

Cyclodextrin (CD) is a circular oligosaccharide composed of α-D(+)-glucopyranose units. The CD, which possesses seven glucose units, is called β-CD. They have a three-dimensional funnel-shaped architecture with a narrower rim molded by a hydrogen-bonding network built by primary OH groups (one group per glucose unit), and with a broader rim composed of secondary OH groups (two groups per glucose unit) (Crini, 2014). The two rims of the molecules are hydrophilic, while the interior of their cavity is hydrophobic. It is known that β-CD tends to form inclusion complexes with guest molecules with suitable characteristics of polarity and dimension in aqueous solutions (Silva et al., 2008; Baâzaoui et al., 2016). CD is among the most frequently used host molecule in supramolecular chemistry; this ability has been widely used in food and pharmaceutical studies (Szejtli, 1998; Crini, 2014). It has also been widely used in lithium battery research as a surfactant to effectively disperse solid substances in liquids and as an agent to promote complexation reactions, which is beneficial to material dispersion and molding (Chen et al., 2016).

**Figure 1:**
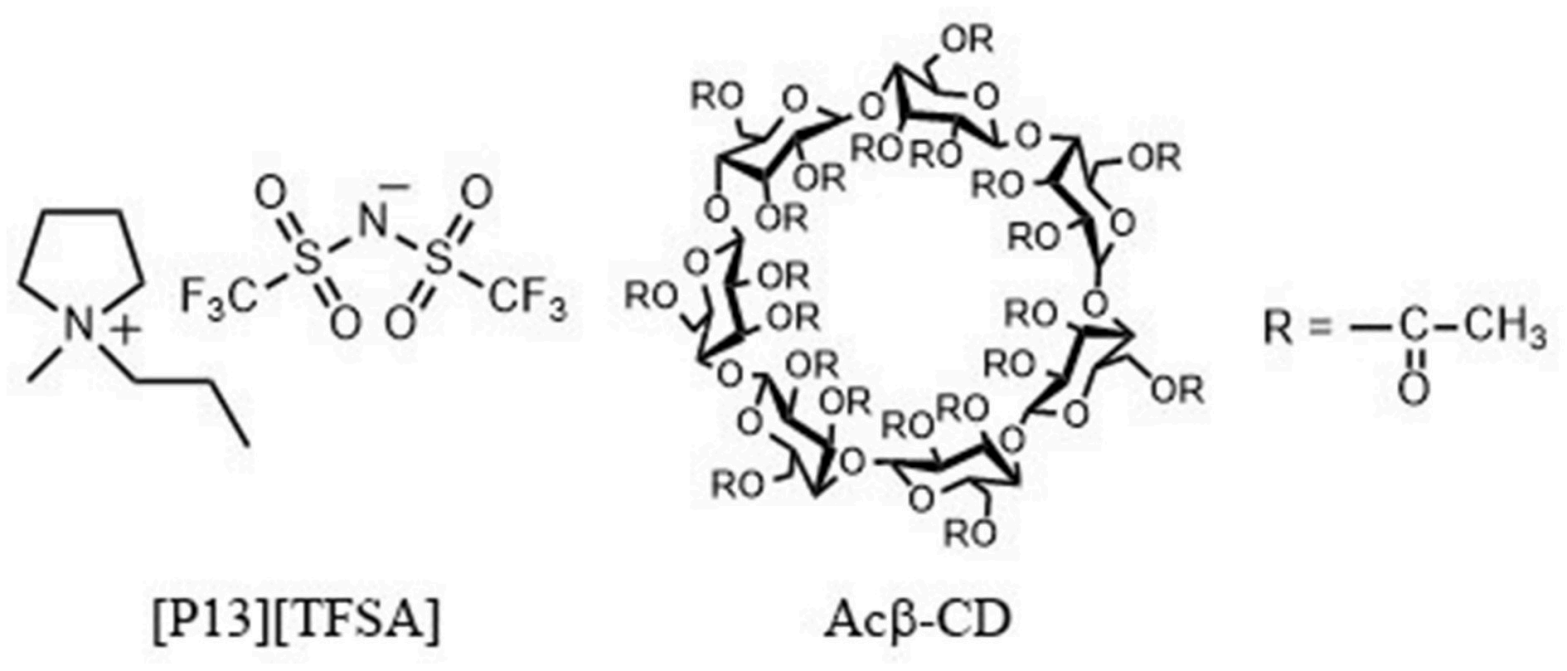
Chemical structures of [C_3_mpyr][TFSA], and Acβ-CD.

Recently, Amajjahe et al. (2008) found that the anion of 1-butyl-3-vinylimidazolium bis(trifluoromethylsulfonyl)amide exclusively formed a host–guest complex with β-CD (Amajjahe and Ritter, 2008; Amajjahe et al., 2008). He et al. (2009) investigated the interaction of hydrophobic ILs and β-CD in detail (He et al., 2009). They found that the imidazolium cation did not interact with β-CD while its long alkyl side chain did. In addition, hydrophobic anions with fluorine atoms could interact with β-CD, and the interaction between the bis(trifluoromethylsulfonyl)amide (TFSA) anion and β-CD was stronger than those of BF_4_ and PF_6_ anions. These results prompted us to investigate the effect of β-CD on the physicochemical properties of ILs, and we expected that the anion trap ability of β-CD would contribute to the enhancement of the Li-ion conductivity and the improvement of the electrochemical stability. In this study, a pyrrolidinium-based IL with a TFSA anion, *N*-methyl-*N*-propylpyrrolidinium bis(trifluoromethylsulfonyl)amide ([C_3_mpyr][TFSA]) (see Figure 1), was used as the electrolyte solution. Its LiTFSA composites were prepared and mixed with different amounts of β-CD, and their physicochemical and electrochemical properties were evaluated.

## Experimental

### Materials

*N*-Methylpyrrolidine (Tokyo Chemical Industry Co., Ltd., > 98.0%), 1-chloropropane (Tokyo Chemical Industry Co., Ltd., > 99.0%), and lithium bis(trifluoromethylsulfonyl)amide (LiTFSA) (Kishida Chemical Co., Ltd., 99.0%) were purchased. *N*-Methylpyrrolidine and 1-chloropropane were purified by distillation *in vacuo* prior to use. Triacetyl-β-cyclodextrin (Acβ-CD) (Tokyo Chemical Industry Co., Ltd., > 97.0%) (see Figure 1) was used after drying.

[C_3_mpyr][TFSA] was prepared as follows. *N*-Methyl-*N*-propylpyrrolidinium chloride ([C_3_mpyr][Cl]) was synthesized according to a previously published procedure (Laus et al., 2008). [C_3_mpyr][Cl] and LiTFSA were separately dissolved in deionized water. LiTFSA aq. was added dropwise to [C_3_mpyr][Cl] aq. The resulting liquid was purified by washing repeatedly with deionized water until no residual chloride was detected with the use of AgNO_3_ aq. [C_3_mpyr][TFSA] was obtained as a colorless liquid at room temperature and characterized by ^1^H NMR, fast atom bombardment mass spectrometry (FAB-MS), and elemental analysis. ^1^H NMR (CD_2_Cl_2_, 300 MHz): δ (ppm) = 3.50 (2H, ddd, *J* = 10.22, 5.58, 3.01 Hz), 3.26 (1H, dt, *J* = 8.59, 4.04 Hz), 3.04 (1.5H, s), 2.27 (2H, s), 1.83 (1H, tt, *J* = 12.20, 5.61 Hz), 1.06 (1.5H, t, *J* = 7.39 Hz). MS (FAB^+^): m/z 128.2 [M], 536.4 [2M+X]^+^, MS (FAB^−^): m/z 280.0 [X], 688.0 [M+2X]^−^. Anal. Calcd. for C_10_H_18_F_6_N_2_O_4_S_2_ (%): C, 29.4; H, 4.44; N, 6.86; S, 15.7; Found (%): C, 29.2; H, 4.42; N, 6.78; S, 16.1.

A given amount of LiTFSA was dissolved in [C_3_mpyr][TFSA] [IL:LiTFSA = 18 : 1 (molar ratio)], and then a given amount of Acβ-CD was added into IL/LiTFSA composites at molar ratios LiTFSA:Acβ-CD = 1.0 : 0.5, 1.0 : 1.0, and 1.0 : 1.5. Four kinds of samples were prepared to investigate the effect of Acβ-CD on the properties of the IL electrolyte. Their composites are abbreviated as the molar ratio of Acβ-CD. For example, the abbreviation of [C_3_mpyr][TFSA] : LiTFSA : Acβ-CD = 18 : 1 : 1.5 is Acβ-CD1.5. These mixtures were stirred at 60°C for 24 h.

### Measurements

Fourier-transform infrared (FT-IR) measurements were performed on a Nicolet 6700 (Thermo Fisher Scientific) by using KRS-5.

^1^H and ^19^F NMR measurements were carried out with a Bruker Avance III HD 400 MHz at 25°C. Thermogravimetric analysis was conducted using a TG-DTA instrument (TG/DTA7200, Hitachi High-Technologies Corp.) under a nitrogen atmosphere at temperatures ranging from 25 to 500°C at a heating rate of 10°C min^−1^. The thermal behavior was examined using differential scanning calorimetry (DSC) (DSC7020, Hitachi High-Technologies Corp.) at temperatures between −150 and 100°C at a heating/cooling rate of 10°C min^−1^.

Impedance measurements were carried out using a VSP-300 (Bio-Logic Science Instruments) at frequencies ranging from 100 mHz to 1 MHz and temperatures ranging from 80 to −40°C. The temperature was controlled by a constant-temperature oven (SU-642, Espec Corp.). The composites were enclosed in a homemade glass cell having two platinum electrodes. The measurements were carried out by maintaining the cells at each temperature for 30 min. Viscosity measurements were carried out using a stabinger viscometer (SVM3000, Anton Paar) and temperature ranging from 80 to 20°C.

Pulse field gradient nuclear magnetic resonance (PFG-NMR) measurements were carried out with a Bruker Avance III HD 400 MHz at 80°C for ^1^H, ^7^Li, and ^19^F nucleus. The ILs were filled into 5-mm NMR tubes, which were sealed. The measurements were carried out in 16 gradient steps per diffusion experiment. The gradient strength was 1,700 G cm^−1^. The diffusion coefficients were calculated from the peak integration attenuation according to Equation 1 (Tanner and Stejskal, 1968):

(1) $$\begin{array}{r} A\;=\;A_{0}-\text{exp}\left[{\left(\gamma \delta \text{G}\right)}^{2}\text{D}\left(\Delta -\frac{\delta }{3}\right)\right] \end{array}$$

where *A* is the signal at a certain gradient (*G*), *A*_0_ is the signal at a gradient of 0, δ is the width of the gradient pulse, Δ is the diffusion time, *D* is the diffusion coefficient, and γ is the gyromagnetic ratio of the nuclei.

Linear sweep voltammetry (LSV) measurements were carried out by using a VSP-300 (Bio-Logic Science Instruments) in the potential range of −0.2 and 6 V at 60°C at a scan rate of 1.0 mV s^−1^. Li foils were used as the reference and counter electrodes, while Ni and Pt plates were used as working electrodes in the potential ranges of −0.2–3.0, and 3.0–6.0 V, respectively. The electrodes were separated by a glass filter to prevent short-circuiting. The cyclic voltammetric measurements of [C_3_mpyr][TFSA]:LiTFSA:Acβ-CD = 18:1:1.0 composites were carried out using a VSP-300 (Bio-Logic Science Instruments) in the potential range of −0.25–1.0 V at 25, at a scan rate of 1.0 mV s^−1^, with Li foils as the reference and counter electrodes, and the Ni plate was used as the working electrode. The electrodes were separated by a glass filter to prevent short-circuiting.

## Results and Discussion

### Interaction Between Acβ-CD and TFSA Anion

A given amount of β-CD was initially added into [C_3_mpyr][TFSA] and its LiTFSA mixture. Unfortunately, the IL electrolytes could not dissolve β-CD at any concentration. β-CD possesses hydroxyl groups, which form hydrogen bonds. The Lewis basicity of the TFSA anion is weak, and the TFSA anion cannot break the hydrogen bond. In fact, ILs with anions such as chloride and acetate, which exhibit a stronger Lewis basicity, can dissolve cellulose (Ohno and Fukaya, 2009) as such anions interact with the hydroxyl groups of cellulose because of the strong electron-donating ability. The TFSA anion could not dissolve even oligosaccharides. Therefore, Acβ-CD was used in this study instead of β-CD. A given amount of Acβ-CD was added into the IL electrolytes. [C_3_mpyr][TFSA] with a weak Lewis-base anion could dissolve Acβ-CD, which has an acetyl group instead of a hydroxyl group.

**Figure 2:**
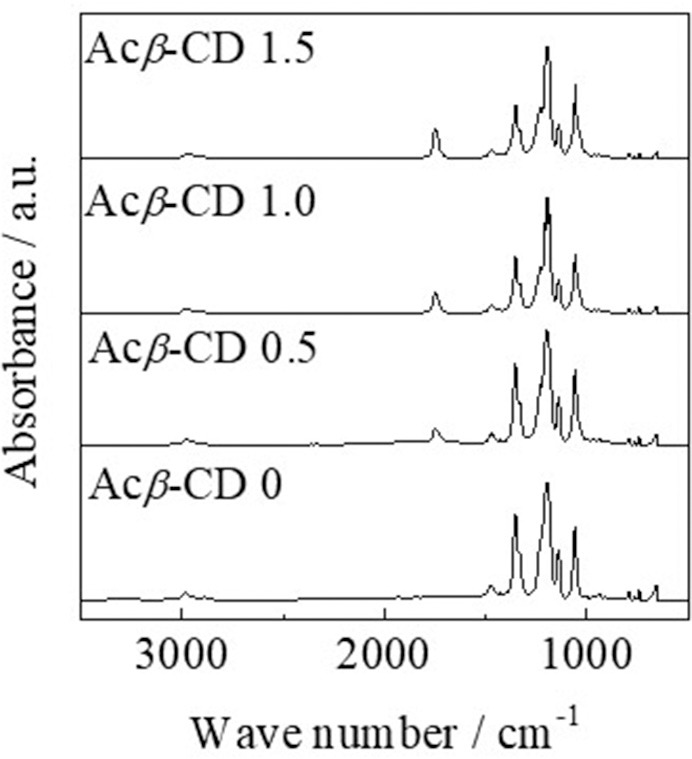
FT-IR spectra of [C_3_mpyr][TFSA]/LiTFSA composites and their Acβ-CD composites.

FT-IR measurements were conducted, and each peak was assigned according to the literatures (Liu et al., 2009; Roy et al., 2016; Li et al., 2017; Wu et al., 2017). Figure 2 presents FT-IR spectra of [C_3_mpyr][TFSA]/LiTFSA and [C_3_mpyr][TFSA]/LiTFSA/Acβ-CD composites. The FT-IR spectrum of [C_3_mpyr][TFSA]/LiTFSA exhibits characteristic peaks for C-H stretching, CH_2_ bending, and S = O stretching bands etc. The peaks in the range from 2,978 to 2,882 cm^−1^ can be assigned to the C-H stretching and CH_2_ bending modes. In the case of TFSA anion, the peaks of S = O stretching band and C-SO_2_-N bond are observed at 1,349 and 1,136 cm^−1^, respectively. In addition, CF_3_ symmetric stretching modes are located in 1,195 cm^−1^ and 1,056 cm^−1^. For the spectrum of [C_3_mpyr][TFSA]/LiTFSA/Acβ-CD, a new peak is observed at 1,746 cm^−1^, which is assigned to C = O stretching mode for acetyl group, and the absorbance increases with increasing the Acβ-CD amount, indicating that the composites are formed by mixing [C_3_mpyr][TFSA]/LiTFSA and Acβ-CD.

**Figure 3:**
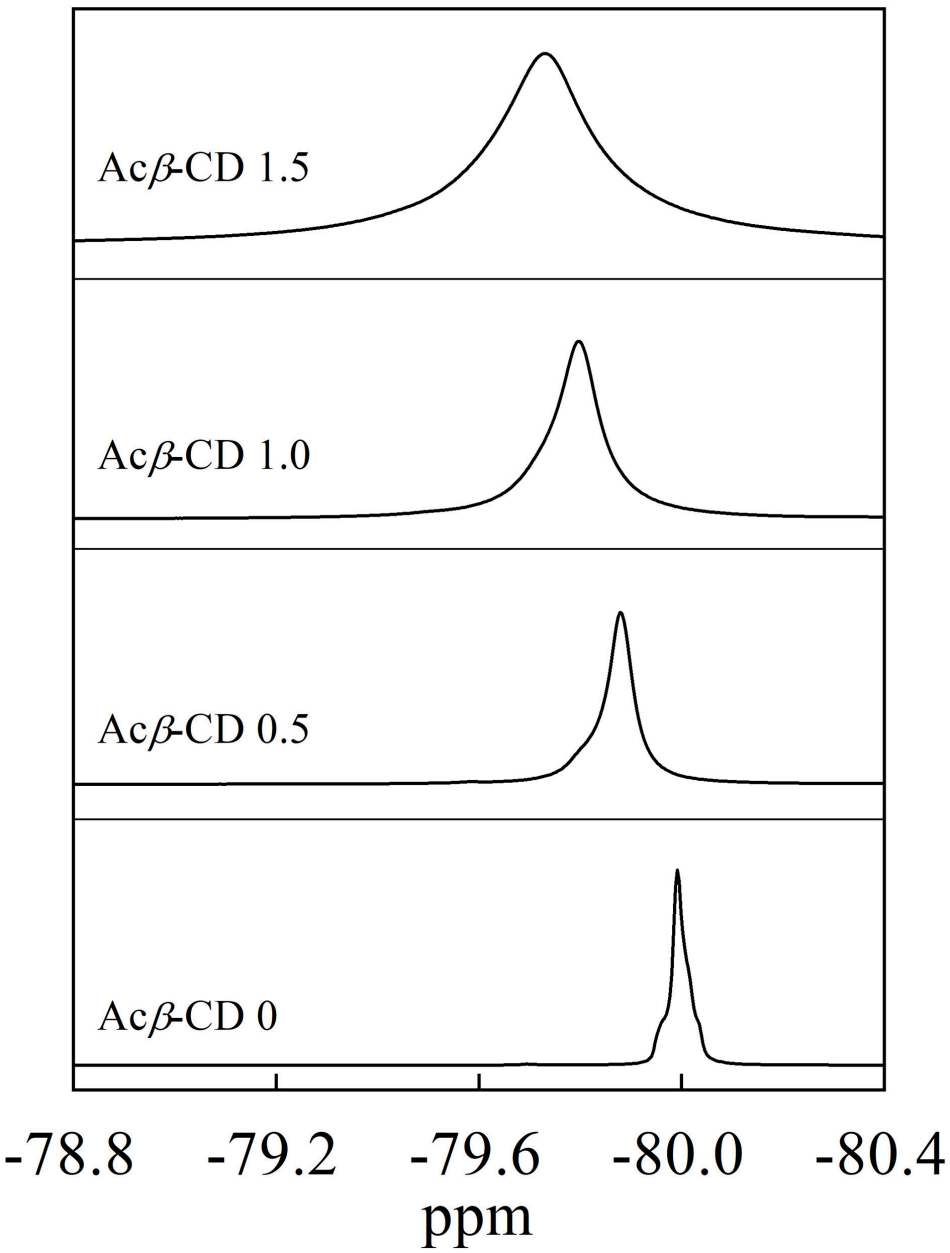
^19^F NMR spectra of [C_3_mpyr][TFSA]/LiTFSA composites and their Acβ-CD composites.

^19^F NMR measurements were carried out to investigate the interaction between Acβ-CD and the TFSA anion. Figure 3 presents ^19^F NMR spectra of the CF_3_ group in the TFSA anion for the [C_3_mpyr][TFSA]/LiTFSA and [C_3_mpyr][TFSA]/LiTFSA/Acβ-CD composites. The chemical shifts of the CF_3_ group of the TFSA anion are −79.73, −79.80, −79.88, and −79.99 ppm for the Acβ-CD 1.5, 1.0, 0.5, and 0 composites, respectively. Zhang et al. (2014) performed ^19^F NMR measurements of 1-ethyl-3-methylimidazolium bis(trifluoromethylsulfonyl)amide to detect the host–guest interaction between the β-CD and TFSA anion (Zhang et al., 2014). As the molar ratio of CD increases, downfield shifts for the fluorine atom of the CF_3_ group in the TFSA anion are observed because of the formation of the complex for CD and the TFSA anion. In all the [C_3_mpyr][TFSA]/LiTFSA/Acβ-CD composites, the CF_3_ group chemically shifts to a lower magnetic field as compared to that of Acβ-CD 0, suggesting that Acβ-CD forms a complex with the TFSA anion.

### Thermal Properties

**Figure 4:**
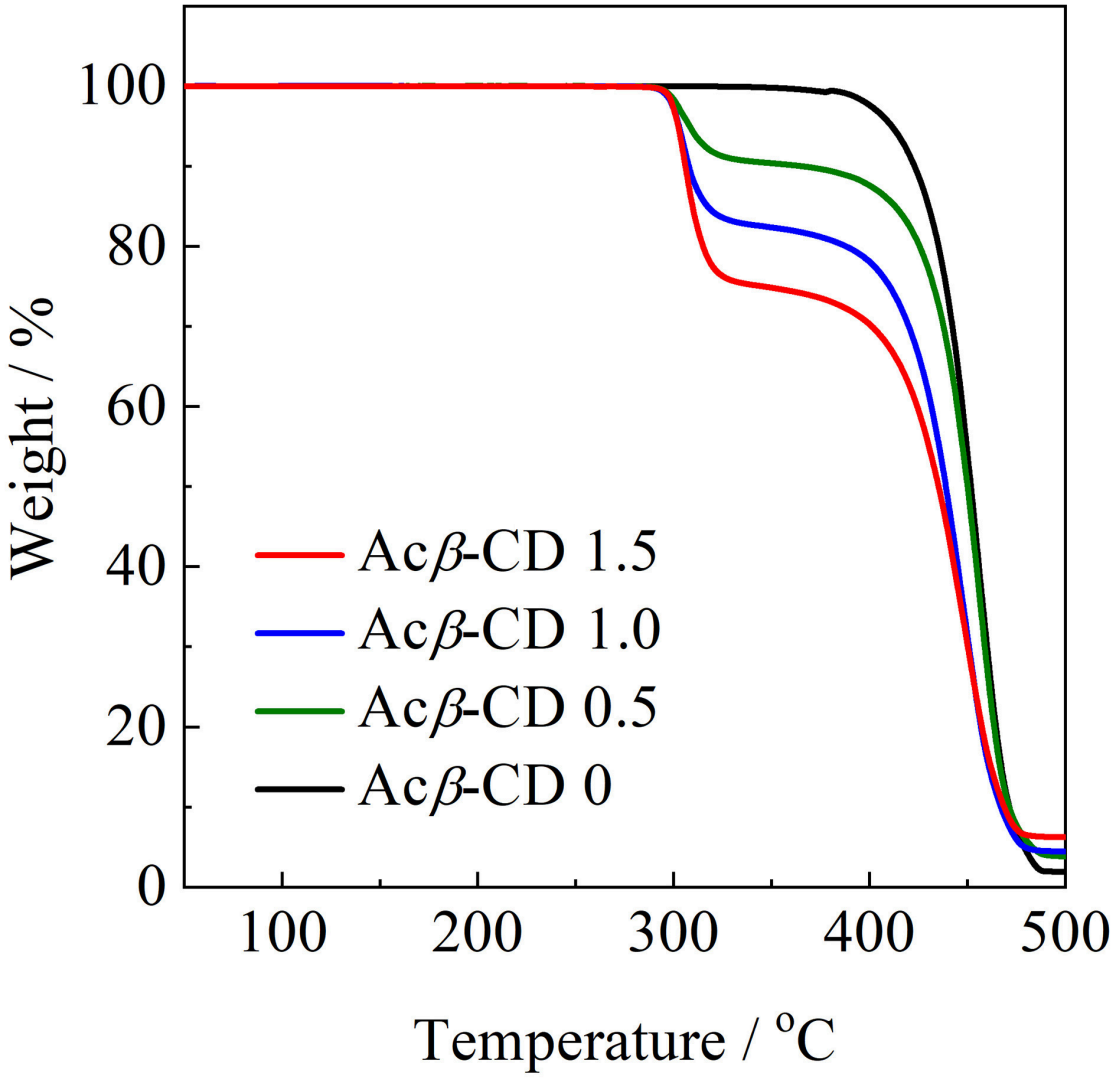
TGA curves of [C_3_mpyr][TFSA]/LiTFSA composites and their Acβ-CD composites.

To evaluate the thermal stability, onset thermal decomposition temperatures (*T*_d_) were measured. Figure 4 shows TGA traces for the [C_3_mpyr][TFSA]/LiTFSA and [C_3_mpyr][TFSA]/LiTFSA/Acβ-CD composites. Acβ-CD 0 shows a *T*_d_ value at 386°C. Pyrrolidinium-based ILs with the TFSA anion are known to exhibit a higher thermal stability, and the *T*_d_ value of Acβ-CD 0 is consistent with the literature value (Yang et al., 2014). All the composites with Acβ-CD show similar *T*_d_ values, and their *T*_d_ values are about 300°C. This is due to the decomposition of Acβ-CD. The amount of weight loss is consistent with the amount of added Acβ-CD in the IL electrolytes.

**Figure 5:**
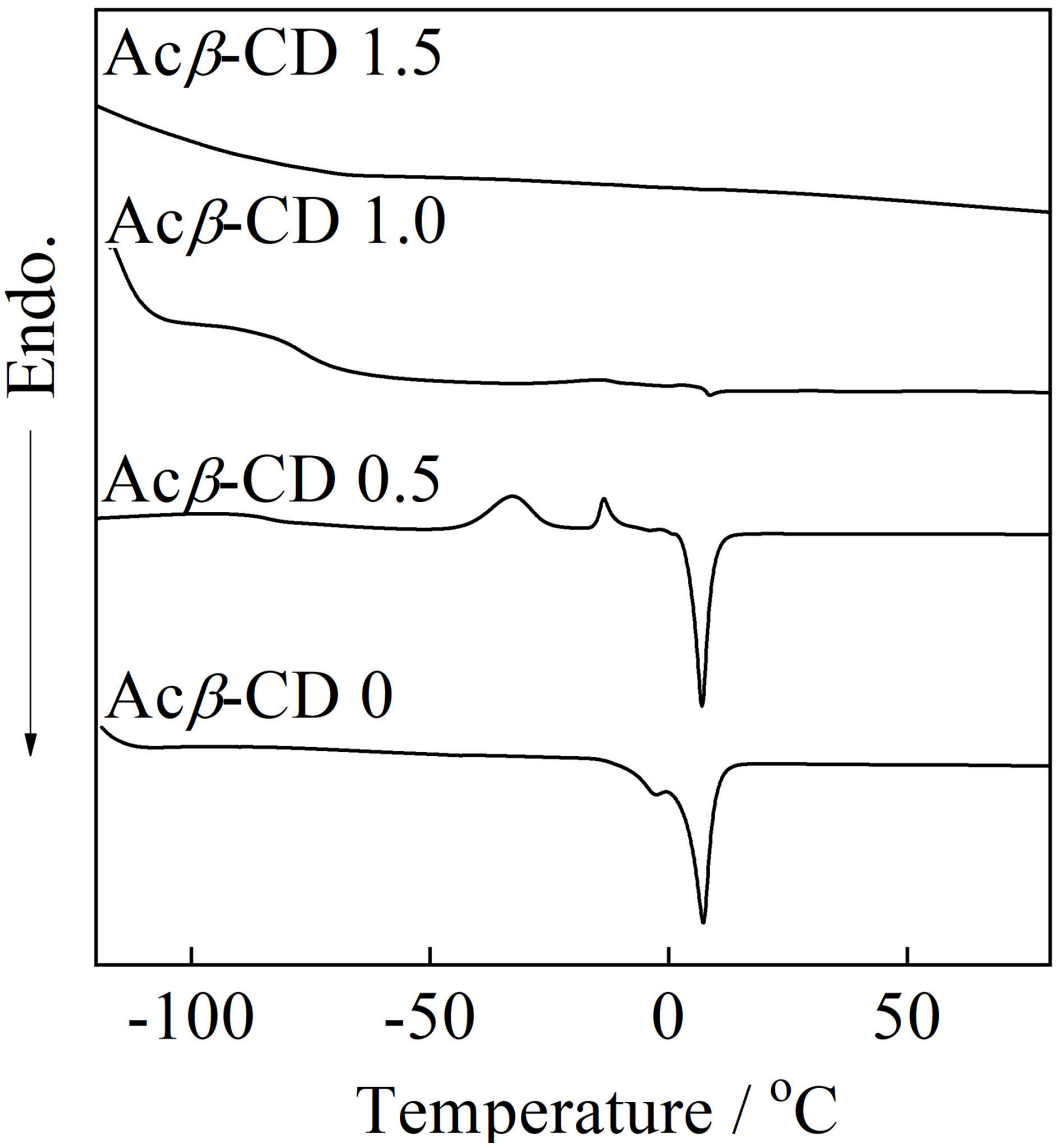
DSC charts of [C_3_mpyr][TFSA]/LiTFSA composites and their Acβ-CD composites.

DSC traces of the composites are presented in Figure 5. Acβ-CD 0 exhibits a melting point (*T*_m_) of 8.0°C, which is consistent with the literature value (Wu et al., 2011). When the addition amount of Acβ-CD is 0.5 in the molar ratio, the glass transition temperature (*T*_g_), two crystallization temperatures, and *T*_m_ are observed at −91, −33, −19, and 7.1°C, respectively. Acβ-CD 0.5 exhibits no crystallization temperature upon the cooling scan. In addition, the *T*_m_ value of Acβ-CD 0.5 slightly decreases as compared to that of Acβ-CD 0. The crystallization temperature and *T*_m_ cannot be observed in the composites in which the added amount of Acβ-CD is larger than the amount of Li salt in the molar ratio. Acβ-CD 1.0 and 1.5 exhibits *T*_g_ only and maintains low values below −79°C. These results suggest that the interaction between the Acβ-CD and TFSA anion prevents the crystallization of the IL.

### Conductivity and Viscosity

**Figure 6:**
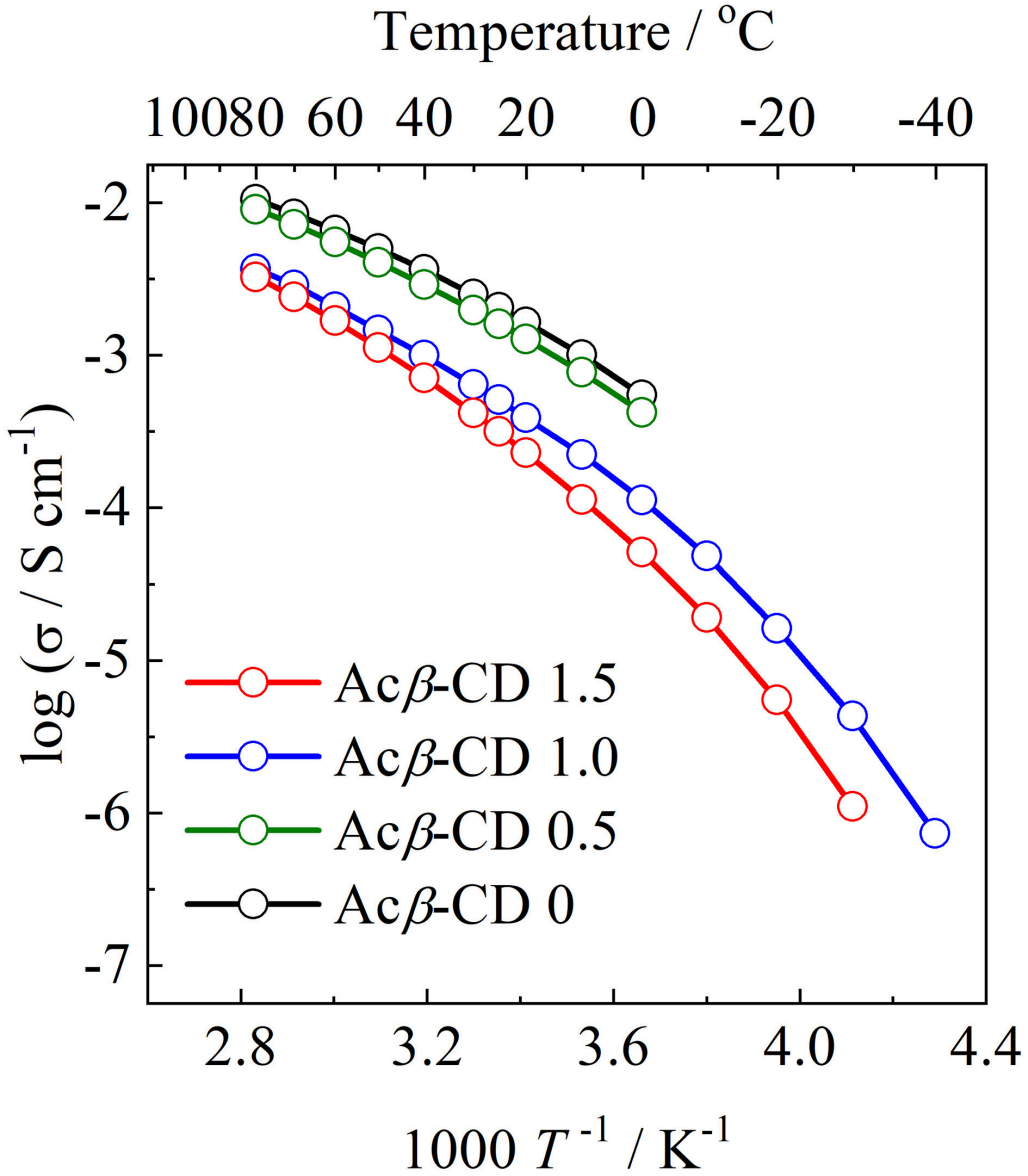
Arrhenius plots of ionic conductivities for [C_3_mpyr][TFSA]/LiTFSA composites and their Acβ-CD composites.

**Figure 7:**
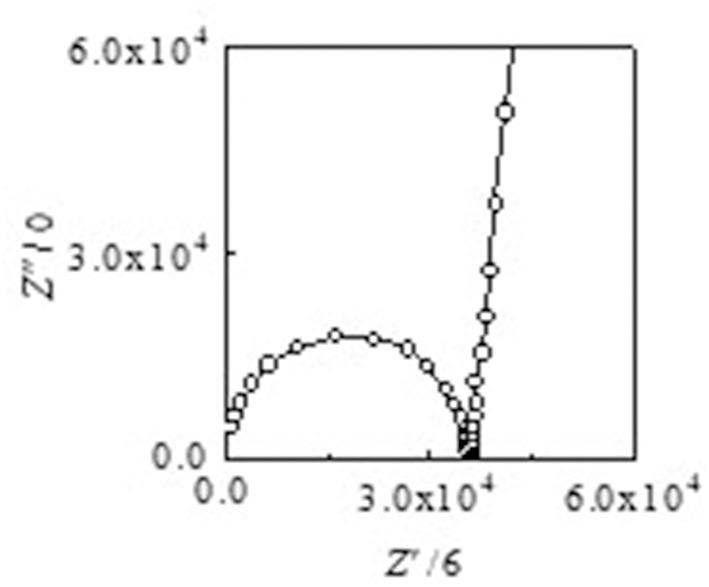
A typical Nyquist plot for Acβ-CD 1.5 at 0°C.

Arrhenius plots of the ionic conductivities for the composite electrolytes are presented in Figure 6. Figure 7 exhibits a typical Nyquist plot for Acβ-CD 1.5 at 0°C. The ionic conductivity values were calculated from the touchdown point on the *Z*′-axis which exhibits the resistance of the compound. For Acβ-CD 0 and 0.5, the conductivity values were not obtained by means of our impedance apparatus, because of the crystallization of the electrolytes below 0°C, which is consistent with the DSC results. According to the DSC results, as Acβ-CD 1.0 and 1.5 are liquid in a wide temperature range, they exhibit a higher ionic conductivity even below 0°C, as shown in Figure 5. The ionic conductivities of the Acβ-CD 1.5, 1.0, 0.5, and 0 composites are 3.2 × 10^−4^, 5.1 × 10^−4^, 1.6 × 10^−3^, and 2.1 × 10^−3^ S cm^−1^ at 25°C, respectively. The addition of Acβ-CD results in the decrease in the conductivity value, ascribable to the formation of an inclusion complex between Acβ-CD and the TFSA anion. This complex decreases the diffusivity of the component ions, thus decreasing the ionic conductivities. Roy and Roy (2017) used trihexyltetradecylphosphonium chloride as an IL, where a similar decrease in conductivity was observed as the amount of CD increased. The decrease in conductivity will be due to the encapsulation of guest molecules in the hydrophobic cavity of CD (Roy and Roy, 2017).

**Figure 8:**
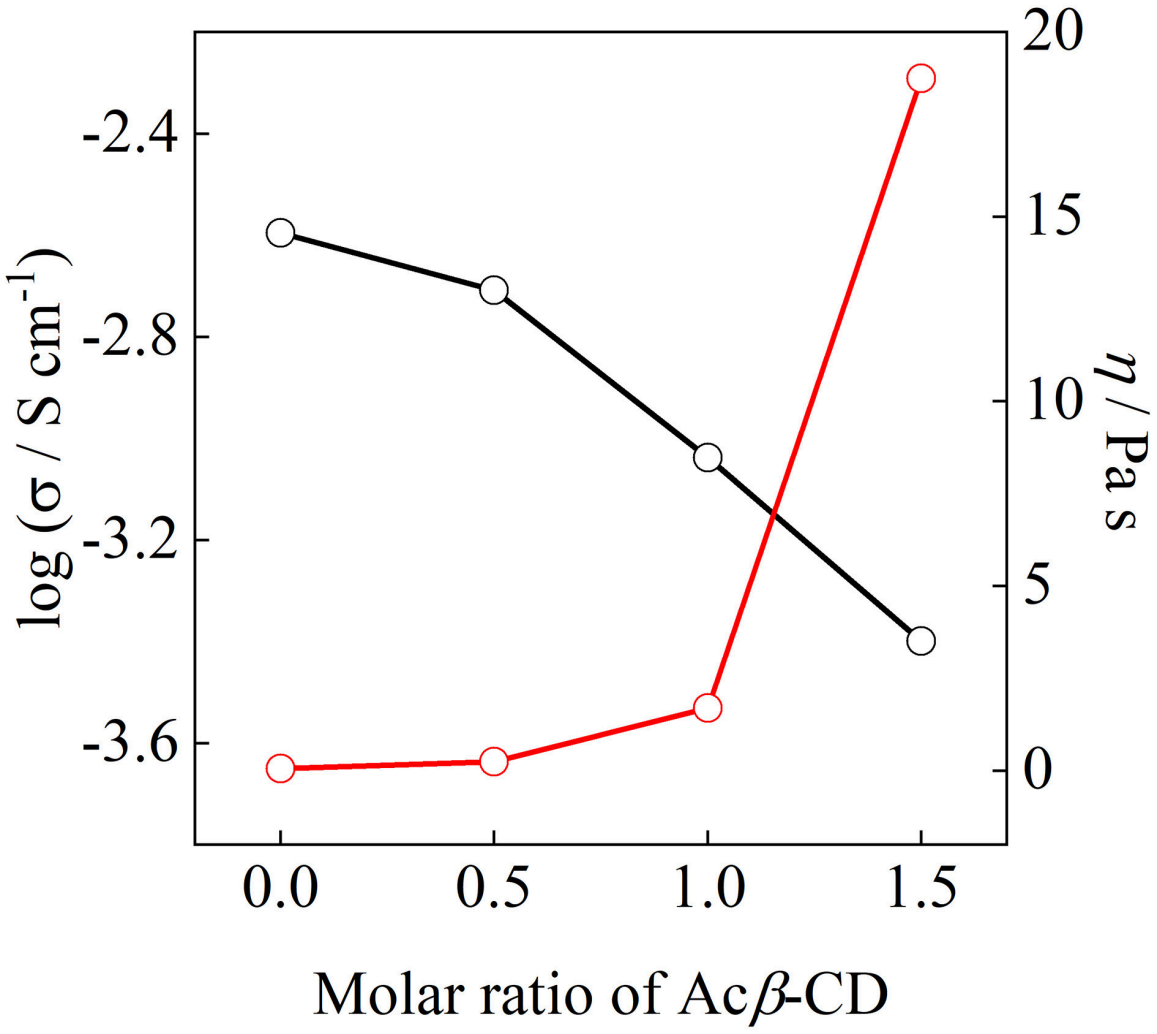
Correlation between ionic conductivity and viscosity for [C_3_mpyr][TFSA]/LiTFSA composites and their Acβ-CD composites.

Figure 8 shows the ionic conductivity at 25°C and viscosity at 30°C as a function of the molar ratio of Acβ-CD. The ionic conductivity monotonously decreases with the Acβ-CD content as mentioned above. The viscosity values of the Acβ-CD 1.5, 1.0, 0.5, and 0 composites are 19,000, 1,700, 250, and 60 mPa s at 30°C, respectively. The viscosity values increase steeply as Acβ-CD is added to the composites. In addition, the conductivities and viscosities are inversely proportional (Salminen et al., 2007). The viscosity increases with CD concentration probably because of the IL and CD interactions and solvation (Roy et al., 2016). Thus, it is considered that the ionic conductivities decrease because of the increase in the viscosities of the composites.

### Diffusion Coefficients

**Figure 9:**
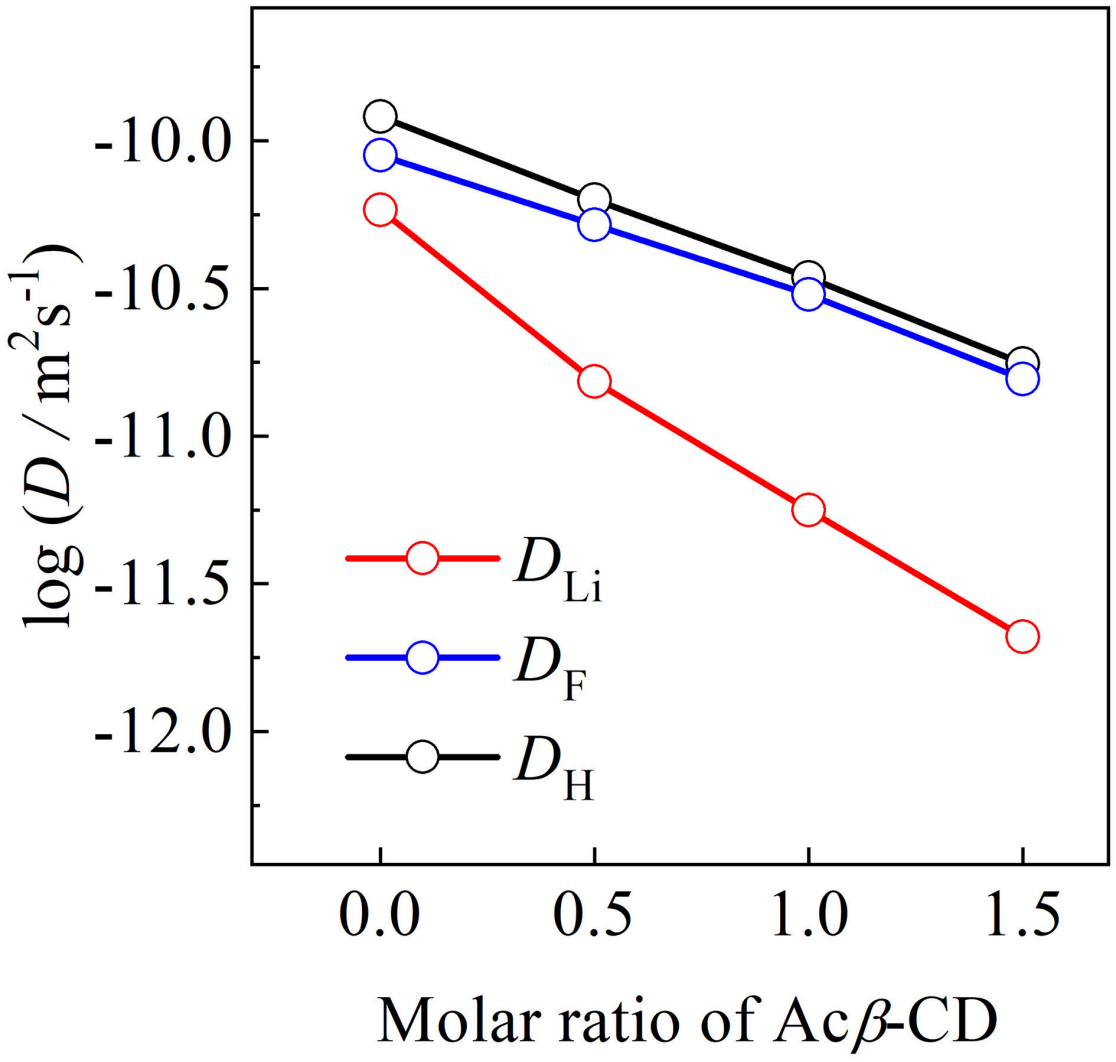
Diffusion coefficients of [C_3_mpyr][TFSA]/LiTFSA composites and their Acβ-CD composites as a function of mole ratio of Acβ-CD.

The self-diffusion coefficients of C_3_mpyr^+^ (*D*_H_), Li^+^ (*D*_Li_), and TFSA^−^ (*D*_TFSA_) for the [C_3_mpyr][TFSA]/LiTFSA and [C_3_mpyr][TFSA]/LiTFSA/Acβ-CD composites were determined by means of PFG-NMR at 80°C, as shown in Figure 9. The *D*_H_ and *D*_TFSA_ values of these composites are almost the same at 80°C, while the *D*_Li_ value is lower than those of the *D*_H_ and *D*_TFSA_ values. The increase in the Acβ-CD content induces a large difference between the *D*_Li_ value and other values. The apparent lithium transfer number (*t*_Li+_) was calculated from the diffusion coefficient values using Equation (2):

(2) $$\begin{array}{l} t_{{\text{Li}}^{+}}=\;\frac{D_{\text{Li }}}{D_{\text{Li }}+\;D_{\text{TFSA }}+D_{\text{H }}} \end{array}$$

Unlike electrochemical techniques, the diffusion coefficients obtained using the PFG-NMR mwcies but also from non-ionic species (Horiuchi et al., 2017). The $t_{\text{Li}}^{+}$ values of the Acβ-CD 1.5, 1.0, 0.5, and 0 composites were 0.06, 0.08, 0.12, and 0.22, respectively. The decrease in the $t_{\text{Li}}^{+}$ values with the increase in Acβ-CD content suggests that an inclusion complex will be formed between LiTFSA and Acβ-CD. In addition, Acβ-CD would form an inclusion complex with not only LiTFSA but also the aggregation including a Li cation, similar to the combination of two TFSA anions and one Li cation because the large surface charge density of a Li cation induces the formation of cluster ions (Appetecchi et al., 2016).

### Electrochemical Properties

**Figure 10:**
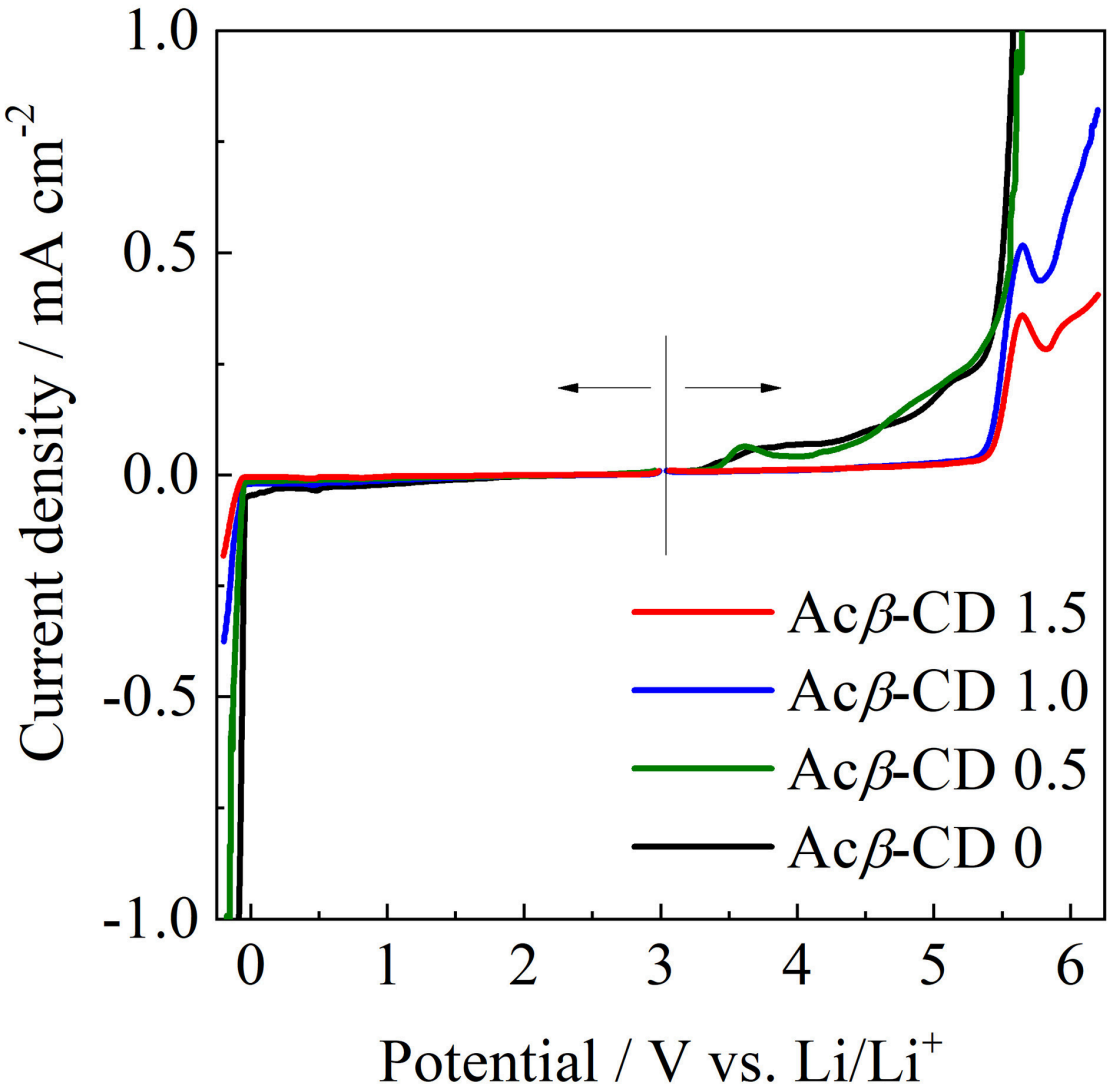
Linear sweep voltammograms of [C_3_mpyr][TFSA]/LiTFSA composites and their Acβ-CD composites.

The electrochemical stabilities of the [C_3_mpyr][TFSA]/LiTFSA and [C_3_mpyr][TFSA]/LiTFSA/Acβ-CD composites were investigated by LSV on a Ni electrode (from 3 to −0.2 V) and Pt electrode (from 3 to 6 V) at 60°C. The LSV results are presented in Figure 10. The electrochemical window (*EW* = *E*_anodic_-*E*_cathodic_) of all the IL electrolytes was determined from the values for the cathodic (*E*_cathodic_) limit at −0.1 mA cm^−2^ and anodic (*E*_anodic_) limit at 0.1 mA cm^−2^. The *EW* value of Acβ-CD 0 is 4.6 V vs. Li/Li^+^ and that of Acβ-CD 0.5 is 4.6 V vs. Li/Li^+^, which is almost the same as that of Acβ-CD 0. The *EW* values are about 5.5 V vs. Li/Li^+^ for both Acβ-CD 1.0 and 1.5 composites. As the addition amount of Acβ-CD increases, the oxidation stability improves. This improvement should be based on the formation of an inclusion complex between Acβ-CD and the TFSA anion because the anodic stability significantly improves, and the cathodic stability is almost the same regardless of the addition of Acβ-CD.

**Figure 11:**
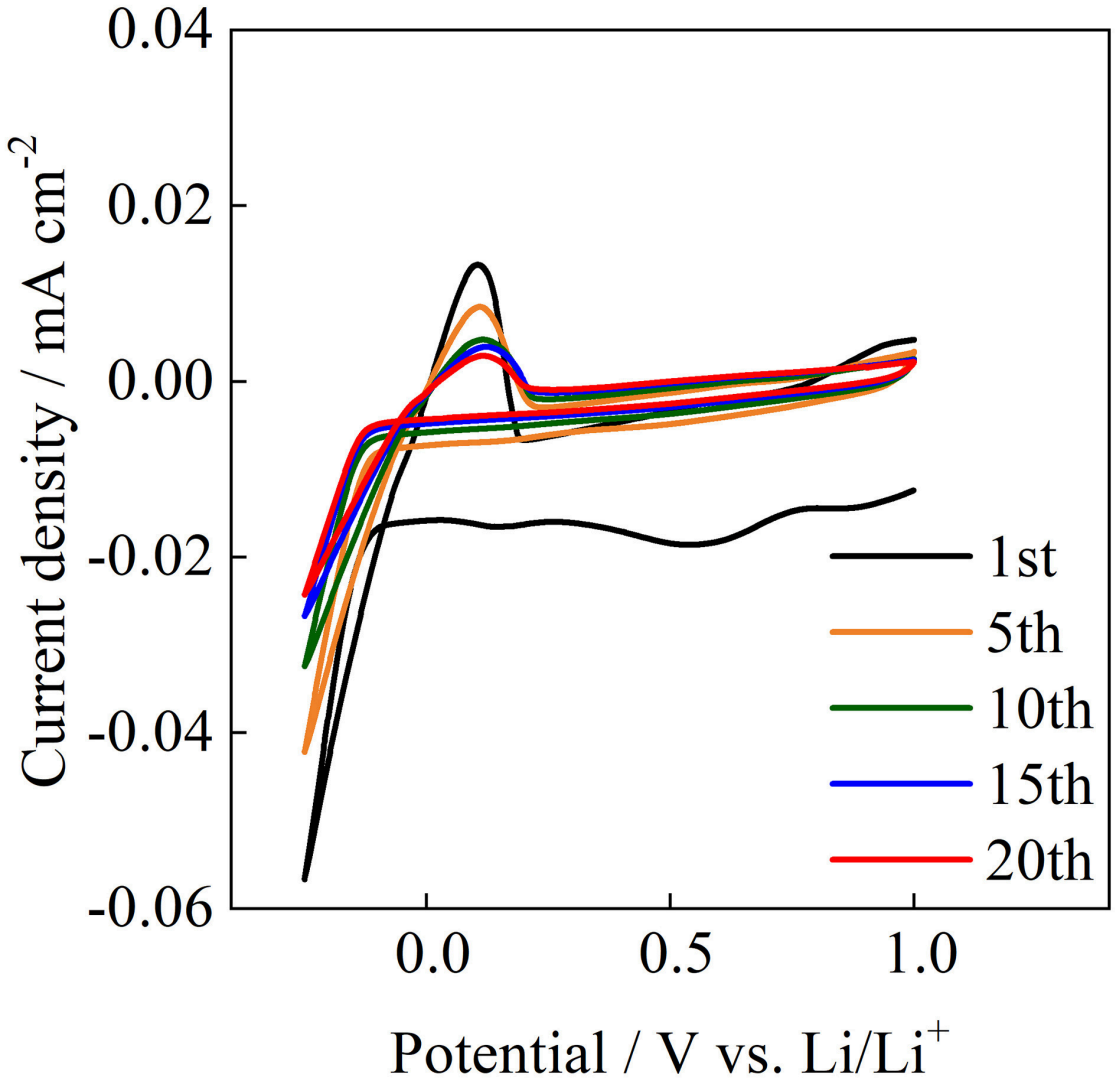
Cyclic voltammograms of Acβ-CD 1.0 at 25°C.

The reversible oxidation and reduction reactions of lithium were examined at room temperature for Acβ-CD 1.0. Figure 11 shows the cyclic voltammogram for Acβ-CD 1.0 on a Ni electrode. Acβ-CD 1.0 exhibits reduction and oxidation peaks for Li at about −0.1 and 0.1 V vs. Li/Li^+^, respectively. The current density decreases with the cycling number from 1st to 10th. After that, the current density maintains a constant value, and stable reversible redox reactions are observed during 20 cycles. At the initial anodic sweep, an anodic current is observed. This behavior is also observed for pyrrolidinium-based ILs (Towada et al., 2015; Horiuchi et al., 2016). In addition, the maximum current density of the anodic peak slightly shifts to a higher potential value with the increase in cycle number. These results suggest that a solid electrolyte interphase film is formed on the Ni electrode surface (Grande et al., 2015), even in the presence of Acβ-CD.

## Conclusions

The effect of Acβ-CD on the properties of [C_3_mpyr][TFSA]/LiTFSA was investigated by means of several techniques. The chemical shift of the CF_3_ group of the TFSA anion shifted to a lower magnetic field with the increase in the Acβ-CD content. With the addition of Acβ-CD to the IL electrolyte, the *T*_m_ of the IL disappeared and the viscosity increased. These results suggest that an inclusion complex is formed between Acβ-CD and the TFSA anion. In contrast, the *t*_Li+_ and *D*_Li_ values decreased with the increase in the Acβ-CD content in the composites. The anodic stability of [C_3_mpyr][TFSA]/LiTFSA was significantly improved after adding a certain amount of Acβ-CD. Li plating and stripping in the [C_3_mpyr][TFSA]/LiTFSA/Acβ-CD composite were repeatedly observed. According to these results, Acβ-CD will be an interesting additive for improving the electrochemical stability of ILs. It is known that there are three kinds of CD, α-CD, β-CD, and γ-CD, which have different cavity sizes. The physicochemical properties of various ILs with different anions could be controlled by choosing suitable CD derivatives.

## Author Contributions

MS and MY-F designed the research. MS prepared the samples and measured the properties. MS and NK carried out the NMR measurements and the data collection. YT, MR, and MY-F participated in the data analysis. MS and MY-F wrote the manuscript.

### Conflict of Interest Statement

The authors declare that the research was conducted in the absence of any commercial or financial relationships that could be construed as a potential conflict of interest.
